# Effectiveness of concomitant use of green tea and polyethylene glycol in bowel preparation for colonoscopy: a randomized controlled study

**DOI:** 10.1186/s12876-020-01220-3

**Published:** 2020-05-13

**Authors:** Zong Hao, Lifeng Gong, Qiang Shen, Huipeng Wang, Shaowen Feng, Xin Wang, Yuankun Cai, Jun Chen

**Affiliations:** 1 Department of General Surgery, The Fifth People’s Hospital of Shanghai, Fudan University, Shanghai, 200240 China; 2 Department of Endoscopic Center, The Fifth People’s Hospital of Shanghai, Fudan University, Shanghai, 200240 China; 3 Department of Colorectal Surgery, Peking University International Hospital, Beijing, 102206 China

**Keywords:** Green tea, Polyethylene glycol, Bowel preparation, Adverse reaction, Colonoscopy

## Abstract

**Background:**

Polyethylene glycol solution (PEG) is widely used for bowel preparation prior to colonoscopies. However, patients often exhibited adverse events as nausea, vomit and distention due to its uncomfortable tastes and potential side affects. This study aimed to evaluate the effectiveness and safety of concomitant use of green tea (GT) with PEG in bowel preparation prior to colonoscopy.

**Methods:**

This was a prospective, randomized controlled study. It was conducted at an outpatient setting of colorectal surgery in a tertiary hospital. Patients aged 18 through 80 who were scheduled to undergo colonoscopy between August 2015 and February 2016 were randomly assigned into two groups, admitting either 2 L-PEG solutions with 1 L GT liquids or 2 L-PEG solutions only for bowel preparation. Admitted doses of PEG solutions, taste evaluation, adverse reactions (nausea and vomiting, distention and abdominal pain) were investigated by questionnaires. The bowel cleanliness of each patient was evaluated according to the Aronchick indicators.

**Results:**

A total of 116 patients were enrolled in this study (PEG+GT 59, PEG 57). Full compliances were achieved in 93.2% patients of group PEG+GT and 59.6% of group PEG (*p* < 0.001). Mean Aronchick scale between two groups were 2.0 ± 0.9 versus 2.2 ± 0.7 respectively (PEG+GT vs PEG, *p* = 0.296). Rates of adverse events as nausea and vomiting, abdominal pain in bowel preparation were significantly different between two groups (55.9% vs 77.2%, *p* = 0.015 and 13.6% vs 33.3%, *p* = 0.012). Patients in group PEG+GT who have probabilities to receive repeating colonoscopy had a higher willingness to accept PEG+GT again for bowel preparation, compared with PEG group (94.9% vs 57.9%, *p* < 0.001).

**Conclusions:**

Concomitant use of green tea and polyethylene glycol may effectively reduce incidence of adverse events, increase compliances, with comparable bowel cleanliness in bowel preparation.

**Trial registration:**

This trial was retrospectively registered on Feb 1st, 2019 (ChiCTR1900021178).

## Background

Adequate bowel preparation is an important factor that ensures the smooth implementation of colonoscopy. Incomplete preparation of the bowel can directly lead to insufficient exposure of colonic mucosa and may result in incorrect or missed diagnosis of bowel lesions [1–3]. Currently, the bowel preparation prior to colonoscopy has been developed recently from earliest diet preparation and subsequent enema using to oral medication currently [4, 5]. Based on the investigation and the comparison of a large number of clinical studies, the ‘consensus of bowel preparation in colonoscopy’ published by the American Society of Gastrointestinal Endoscopy (ASGE), the American Society of Colon and Rectal surgeons (ASCRS) and the Society of American Gastrointestinal and Endoscopic Surgeons (SAGES) in 2006 [6], suggested that polyethylene glycol electrolyte solution (PEG) is a fast and effective method for bowel cleansing with improved tolerance, which is considered superior to the high-dose laxatives and mannitol that require patient fasting. At present, polyethylene glycol electrolyte powder has become the most common bowel cleanser during the process of bowel preparation. However, during the process of ingestion of PEG solution, the majority of the patients develop adverse reactions, such as nausea, vomiting, abdominal distention and pain, due to its bitter taste. This results in poor tolerance from patients which lead to incomplete administration of the polyethylene glycol electrolyte solution and in turn influence the efficacy of bowel preparation.

Green tea has played a significant role in the Chinese traditional culture and lives. Recently, more studies showed its potential benefits in the fields of anti-cancer effects [7–9], protection of cardiovascular system and improvement of neurological and spiritual disorders [10–12]. Few studies investigated its inhibition of bacterial infection [13]. However, scant data focused on its protective impact on gastrointestinal system and whether green tea using as a flavor could reduce side effects during bowel preparation remains unknown. This study aimed to investigate potential effects of green tea on bowel preparation prior to colonoscopy through combination of PEG.

## Methods

### Study design

This study was a prospective, randomized, endoscopist-blinded study. The study protocol was approved by the Ethics Committee of The Fifth People’s Hospital of Shanghai Fudan University (No.2015038). Written informed consent was obtained from all individual participants included in the study. It was conducted at an outpatient setting.

### Study subjects

Patients age 18 through 80 years old scheduled to undergo colonoscopy by our two assigned endoscopists between August 2015 and February 2016 were enrolled consecutively. Indications of colonoscopy included routinely screening for colorectal cancer or surveillance of polyps. Exclusion criteria were as follows: uncontrollable acute or intractable chronic infections or unhealed wounds, presented with gastrointestinal bleeding, gastrointestinal stenosis or bowel obstruction, with comorbidities as severe cardiovascular, liver, kidney or hematopoietic system failure, histories of abdominal surgery. Patients greater than 80, less than 18, pregnant, mentally disabled, have no habit of drinking green tea or cannot tolerate taste of green tea were also excluded.

### Randomization and group allocation

Patients who consented to participate our study and fulfilled the inclusion criteria were randomly assigned into either the experimental or control group. Randomization with an allocation ratio of 1:1 was generated by computerized randomization program with permuted blocks of 10.

All patients were instructed to adhere to a low-residue diet for 3 days. Patients who were allocated into the PEG+GT group (experimental group) received a total of 2 L PEG solution plus 1 L GT liquids, while patients in PEG (control group) administered 2 L PEG solution plus 1 L clear water for the bowel preparation. All patients were not allowed to consume anything but water after 10 pm the day before the exam. They were instructed to intake the medication between 6 and 11 am on the day of colonoscopy. All colonoscopies were performed between 12 pm and 3 pm. Staff members performing the colonoscopy were not allowed to interact with patients about drug-related activities before or during procedures to ensure the blinding maintenance.

### Study medications

PEG powder (®Shu Taiqing, Staidson Biopharmaceuticals Co. Ltd., China) was packed in a box including 6 bags of reagent A and B respectively. Each bag of reagent A contains 13.125 g of PEG4000 while reagent B consisting 0.1785 g of sodium bicarbonate, 0.3507 g of sodium chloride and 0.0466 g of potassium chloride. A total of three boxes of PEG powder and 2 L clear water were made as PEG 2 L solution. Green Tea leaves (®Meijiawu Longjin) were harvested in February 2015 and packed at a 20 g of one bag, storing in a suitable dried environment. GT liquids were acquired after purifying solution brewed with GT leaves and warm water at a 25 C 40 min earlier.

### Measurements

Demographics such as gender, age and body mass index were recorded. Actual intake dose of PEG solution of each patient was recorded for evaluating the compliance. Measurements of the primary outcome is the rates of completeness of administrated liquids. Questionnaires including taste evaluation, adverse reactions, repeat willingness were acquired from each patient prior to the colonoscopy, delivered by one independent researcher who was blinded of group allocation. Colonoscopy was conducted by two skilled endoscopists who had no knowledge of the grouping of the patients. The bowel cleanliness of each patient was evaluated via Aronchick indicators [14]. With the Aronchick scoring system, the bowel cleanliness is rated with a 5-point grade based on 2 criteria, the proportion of fluid and remnant stool (excellent = 0, good = 1, fair = 2, poor = 3, and inadequate = 4). An effective bowel preparation was defined as being excellent or good on the Aronchick scoring system.

### Statistical methods

The sample size was calculated as a superiority study basing on the normal approximation to the binomial distribution. Using the results from former studies, complete rate of PEG liquids administration was expected to be 70 to 85% [15, 16]. Assuming an 80% complete assumption rate of PEG liquids, based on a 1-sided 2-sample t test, a sample size of 59 subjects per group will have 80% power to detect a treatment difference of 15% with a type I error rate of 5%. Dropout rate was estimated no more than 5%, 60 patients were calculated to be enrolled in each arm.

Stata/SE14.1 software was used for data analysis. All continuous data were expressed as mean ± standard deviation, and t test and/or nonparametric test were conducted. Categorical data were compared using chi-square test and a *P* value of less than 0.05 (*p* < 0.05) was considered to be statistically significant.

## Results

### Patient characteristics

**Fig. 1:**
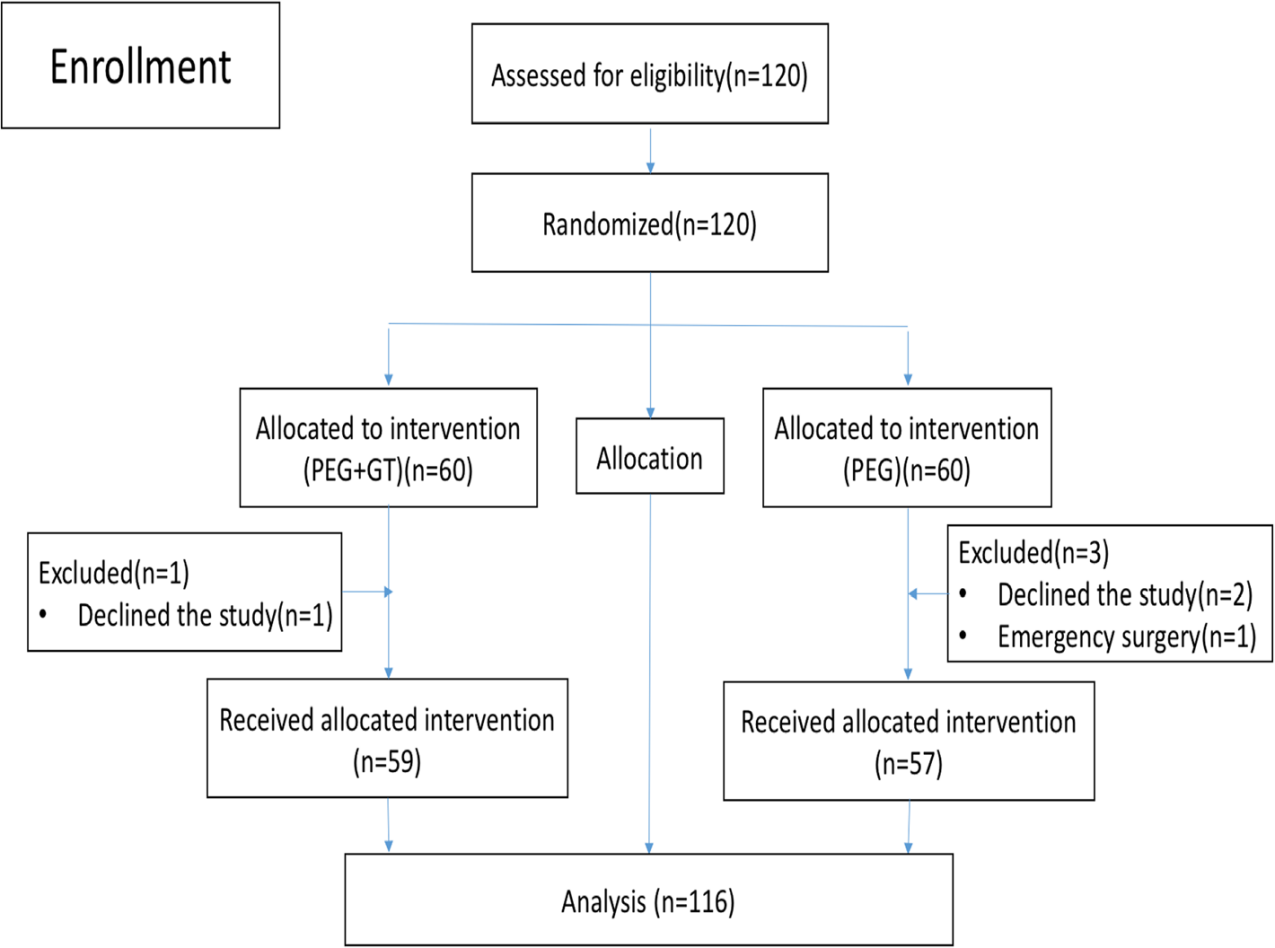
Patient flow. PEG = polyethylene glycol; GT = green tea

Overall 120 patients were randomly assigned to the study with 60 in each group. Three patients refused to receive allocated intervention (2 in PEG group, 1 in PEG+GT group). One case in the PEG group withdrew from the study due to emergency conditions as aggravated abdominal pain who was admitted for surgery. Ultimately, a total of 116 cases completed the study, including 59 cases in PEG+GT group and 57 cases in PEG group (Fig. 1). Characteristics were shown in Table 1. There were no differences regarding sex, age, body mass index and comorbidities and endoscopic findings as polyps and cancer detection between the two groups (*p* > 0.05).

**Table 1:** Demographics of patients in the two groups

Characteristics	PEG+GT Group(*N* = 59)	PEG Group(*N* = 57)	P value
Age(y)(mean ± SD)	54.1 ± 14.2	57.6 ± 11.0	0.142
Male gender(%)	32(54.2)	36(63.2)	0.329
Height (cm)(mean ± SD)	168.6 ± 6.8	166.2 ± 7.7	0.081
Weight (kg)(mean ± SD)	66.4 ± 6.9	65.2 ± 11.2	0.476
BMI (kg/m^2^)(mean ± SD)	23.3 ± 1.7	23.5 ± 3.1	0.709
Comorbidities	15(25.4)	21(36.8)	0.184
Hypertension	13(22.0)	15(26.3)	
Diabetes	2(3.4)	6(10.5)	
Polyp detection	9(15.3)	14(24.6)	0.209
Cancer detection	2(3.4)	5(8.8)	0.268

*PEG* polyethylene glycol, *GT* green tea

### Compliance, tolerance and adverse reactions

As is shown in Table 2, in bowel preparation, rate of patients in PEG+GT group who administered full dose of allocated solution, was significantly higher than the rate in PEG group (93.2% vs 59.6%, *p* < 0.001). Tolerance indicators as the taste evaluation of solution showed that patients who received PEG+GT solution had a larger possibility of satisfaction comparing with patients in the PEG group(*p* < 0.001). Patients in the PEG+GT group exhibited higher willingness to repeat using if they would have a secondary colonic preparation(p < 0.001). Adverse reactions of the patients were recorded. Patients were more probably had nausea, vomit and abdominal pain in PEG group, comparing with the PEG+GT group (*p* < 0.05).

**Table 2:** Comparison of the compliance, tolerance between the two groups of patients

Variable	PEG+GT Group (*N* = 59) (%)	PEG Group (*N* = 57) (%)	*P* value
Complete intake			
Yes	55(93.2)	34(59.6)	0.000
No	4(6.8)	23(40.4)	
Taste evaluation			
Dissatisfaction	2(3.4)	11(19.3)	0.000
Normal	14(23.7)	33(57.9)	
Satisfaction	36(61.0)	11(19.3)	
Highly satisfactory	7(11.9)	2(3.5)	
Repeat willingness	56(94.9)	33(57.9)	0.000
Adverse reactions			
Nausea and vomiting	33(55.9)	44(77.2)	0.015
Distention	36(61.0)	44(77.2)	0.060
Abdominal pain	8(13.6)	19(33.3)	0.012

*PEG* polyethylene glycol*, GT* green tea

### Efficacy

**Fig. 2:**
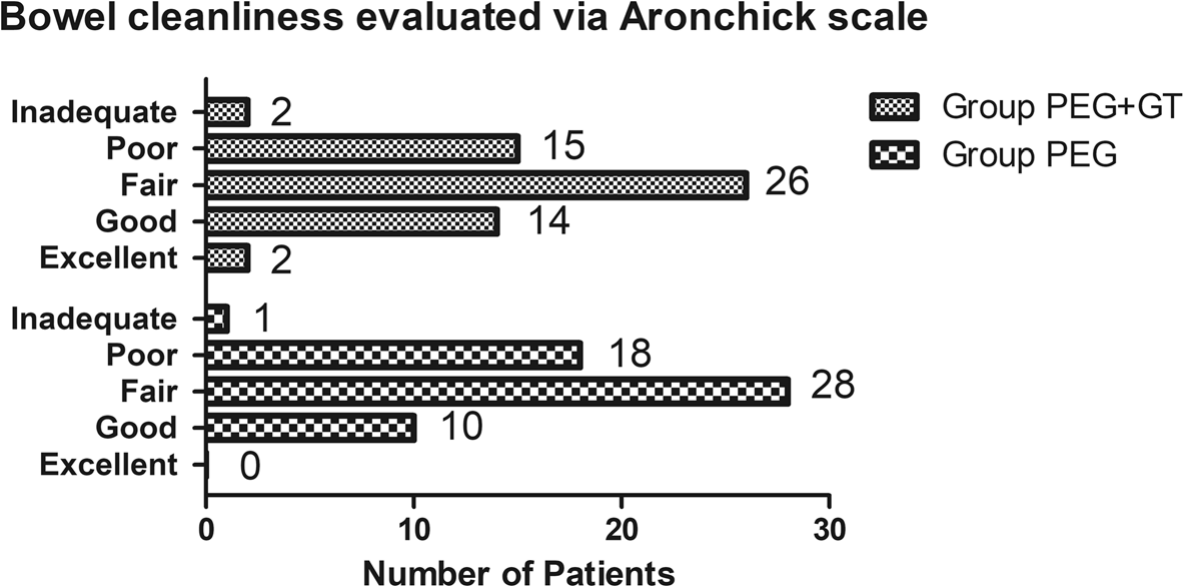
Chart of bowel cleanliness evaluated via Aronchick score system. PEG = polyethylene glycol; GT = green tea

Aronchick indicators were used to evaluate the efficacy of bowel preparation. Sixteen patients in the PEG+GT group acquired an evaluation of cleanliness as excellent or good (Aronchick scale = 0 or 1), comparably 14 in the PEG group (27.1% vs 24.6%, *p* = 0.75) (Fig. 2). The mean Aronchick scores of the two groups were 2.0 ± 0.9 and 2.2 ± 0.7 respectively (PEG+GT vs PEG, *p* = 0.296).

## Discussion

Inability to intake the complete dose of preparation because of poor tolerability may reduce the efficacy of the colonoscopy [17–19]. Nevertheless, due to bitter taste and large volume liquids of PEG, patients tend to develop adverse reactions, such as nausea, vomiting, and abdominal distention during bowel preparation. In our study, more than 2/3 patients in the control group exhibited one or more complications during the preparation, similar as it was reported [20, 21]. Mounts of strategies were used by clinicians to improve compliance of the patients as low-volume PEG preparation [22, 23], split-dose modification [21, 24], flavors using and so on [16, 25]. In our study, green tea was used as a supplement in order to investigate its potential improvement of the tolerability and compliance. Mean actual consumption of the liquids in PEG+GT group were more than the PEG group accordingly. And patients in experimental group showed a stronger willingness to repeat using if they would have to receive a second bowel preparation, comparing with the control group. It is probably because of the nature of the green tea and the habitual green tea drinking in Asian population. As to the side effects during the preparation, patients in PEG+GT group reported significantly less vomit, nausea and pain compared to PEG group. This probably due to its preventing of the digestive tract from inflammation. Hamid, Rahman SU [26, 27] etc. also reported similar results that green tea could inhibit diarrhea and vomit in patients with inflammatory bowel disease (IBD) related or radiological injuries. Its mechanism maybe due to its substance catechin, which can inhibit the synthesis of nitric oxide (NO)-synthase, a bioactive molecule that plays an essential role in inflammation [28].

Besides, all green tea leaves were harvested the year before the study which was considered as brand fresh ones in order to improve the taste and quality of the liquids. Then, these leaves were brewed at a 25 °C water setting for 40 min which was helpful to preserve maximum activities of its substances. Other similar studies using orange or pineapple juice as flavors of the PEG solution also found notable more compliances as well as less adverse reactions in experimental patients. It may due to its potential effects about accelerating the gastric empting, increasing the bowel activities and reduce the discomforts of bowel cleansing [25]. And the change of the acidic taste and lower PH value of the solution were also one indispensable cause.

Regarding the effectiveness of the bowel preparation, validated Aronchick scale was used. It did not show a notable improvement of bowel cleanliness with the addition of GT in this study. One main reason maybe residues in the green tea beverages ingested concurrently during bowel preparation, which impacted the evaluation of cleanliness. Potential changes of osmotic pressure were another consideration with the combination of green tea and PEG in bowel preparation. Less than 5% of tea catechins were absorbed in the small intestine according to the studies of pharmacokinetics of green tea catechins [29, 30]. Tea catechins just passed the small intestine without absorption or recycling. They were then broken down to ring-fission metabolites by colonic bacteria which could potentially raise the osmotic pressure in the bowel, having synergistic actions to PEG [31, 32]. Further researches are needed to discover the mechanisms.

A particular strength of this study is that this is the first study to confirm the clinical efficacy and tolerability of green tea combing with PEG for bowel preparation in Asia. Certainly, future studies of large sample of Westerners need to extend and convince this results since there are less green tea drinking habits in other race.

This study has its intrinsic limitations as part of data were acquired subjectively through questionnaires. And there is no standardized green tea product regarding its origination, process of production, leaves selection which may lead to varieties of green tea beverages. And there is no blood or urine test for evaluating the patients’ internal environment which were possibly affected by the interventions in the two groups.

## Conclusion

Combination of green tea with polyethylene glycol electrolyte powder can effectively reduce the incidence of adverse reactions, increase the compliance of the patients with comparable effects of bowel preparation prior to colonoscopy.

## Data Availability

The datasets used during the current study are available from the corresponding author on reasonable request.

## Abbreviations

PEG: Polyethylene glycol
GT: Green tea
ASGE: American Society of Gastrointestinal Endoscopy
ASCRS: American Society of Colon and Rectal surgeons
SAGES: Society of American Gastrointestinal and Endoscopic Surgeons
